# Dual-Layer Natamycin and Boric-Acid-Reinforced PVA/Chitosan by 3D Printing and Electrospinning Method: Characterization and In Vitro Evaluation

**DOI:** 10.3390/polym17121673

**Published:** 2025-06-17

**Authors:** Büsra Oktay, Fatih Ciftci, Azime Erarslan, Esma Ahlatcıoğlu Özerol

**Affiliations:** 1 Department of Bioengineering, Yildiz Technical University, Istanbul 34210, Turkey; busra.oktay@std.yildiz.edu.tr (B.O.); yilmaz@yildiz.edu.tr (A.E.); 2 Department of Biomedical Engineering, Fatih Sultan Mehmet Vakıf University, Istanbul 34015, Turkey; 3 BioriginAI Research Group, Department of Biomedical Engineering, Fatih Sultan Mehmet Vakıf University, Istanbul 34015, Turkey; 4 Department of Technology Transfer Office, Fatih Sultan Mehmet Vakıf University, Istanbul 34015, Turkey

**Keywords:** 3D printing, antifungal, boric acid, nanofibers, natamycin, wound dressing

## Abstract

This study presents the development and comprehensive characterization of biopolymer-based nanofibrous composites composed of polyvinyl alcohol (PVA), chitosan (CS), boric acid (BA), and a natural antifungal agent natamycin (NAT), designed for therapeutic applications. A dual-layer 3D-fiber composite (PVA/CS/BA_PVA/NAT) was successfully fabricated using a layer-by-layer 3D bioprinting technique and electro-spinning, integrating BA into the core matrix and NAT into the outer layer. Mechanical tests revealed a significantly improved elastic modulus of 763.04 ± 14.54 MPa and the highest ultimate tensile stress (50.45 ± 2.58 MPa) among all samples. Despite a moderate strain at break (11.77 ± 0.49%), the composite preserved sufficient elasticity suitable for biological interfaces. Morphological assessment via SEM confirmed the successful deposition of continuous and bead-free nanofibers, with controlled fiber alignment and reduced average fiber diameters, especially in the BA-incorporated structure. The dual-layered system displayed enhanced uniformity and structural coherence. The drug release analysis demonstrated sustained NAT delivery over a 90 min period. Kinetic modeling showed a high correlation with the Korsmeyer–Peppas model (R^2^ > 0.99), suggesting diffusion-controlled release, supported by the Korsmeyer–Peppas model’s Fickian diffusion exponent. In contrast, zero- and first-order models exhibited weaker fits, underscoring the relevance of a matrix-based release mechanism governed by the layered configuration. Crucially, antifungal assays against *Candida albicans* revealed substantial bioactivity. The PVA/CS/BA_PVA/NAT formulation achieved the largest inhibition zone (1.64 ± 0.13 cm), significantly outperforming single-layer controls such as PVA/CS/BA (1.25 ± 0.08 cm) and PVA/CS_PVA/NAT (1.43 ± 0.08 cm), while neat PVA exhibited no inhibition. These results confirm the synergistic antifungal efficacy of BA and NAT within the dual-layer structure. Together, these findings highlight the potential of the 3D-printed PVA/CS/BA_PVA/NAT composite as a mechanically robust, morphologically optimized, and bioactive platform for antifungal therapy and wound-healing applications.

## 1. Introduction

Human skin, the body’s largest organ, serves as a protective barrier and is colonized by bacteria, viruses, and fungi. Interactions between the skin and environmental factors can lead to various skin diseases [1]. Skin infections occur when pathogens penetrate through wounds or openings in the skin [2,3]. Microorganisms trigger host responses, causing symptoms such as redness, swelling, pain, inflammation, and pus-filled blisters [4,5]. Additionally, a high microbial load can delay wound healing [6]. Fungal infections can be superficial (affecting the skin, mucosa, nails, and hair) or invasive. The main causes of superficial fungal infections include dermatophytes (most commonly *Trichophyton rubrum*), Candida species (with *Candida albicans* being the most prevalent), and *Malassezia* (*Pityrosporum*) fungi [7]. The diagnosis and treatment of fungal infections require a specialized approach depending on the type of infection and the patient’s condition. A wound dressing is an element that creates a physical barrier between the wound and the external environment, protecting the wound from microorganisms and further damage to accelerate the healing process [8]. These dressings can reduce scar tissue formation and inflammation while increasing the rate of epithelialization, which contributes to wound healing and supports the accumulation of ECM [9,10]. Today, modern wound dressings are preferred over traditional ones. These modern dressings are developed to maintain the wound’s moisture balance and provide mechanical and physiological support to the healing process. Therefore, the primary goal of modern wound dressings is not just to cover the wound but to support the healing process. Polyvinyl alcohol (PVA) is a transparent, white, odorless, tasteless, and crystalline synthetic polymer that easily dissolves in water. Its ability to crosslink due to the hydroxyl groups in its structure makes it important for its biocompatibility and biodegradability. With excellent biocompatibility, PVA is widely preferred in the biomedical and pharmaceutical industries due to its good film-forming properties, non-toxicity to the human body, and long-term thermal stability [11]. Chitosan (CS), a deacetylated derivative of chitin, is also widely used in biomaterials due to its biocompatibility, excellent biodegradability, antimicrobial activity, rapid wound healing, and non-toxicity. CS has a unique ability to increase drug absorption in tissues by adhering to the biological environment in drug delivery systems [12]. Boron, in the form of boric acid (BA), is compatible with physiological pH values [13]. BA is preferred for its broad-spectrum antibacterial properties and its ability to support cell proliferation. Many studies have shown that boric acid has strong antimicrobial effects on bacteria, yeasts, and fungi. Additionally, it promotes macrophage migration, increases cell proliferation, and significantly raises the gene expression levels of dermal cells [14]. BA has been found to contribute to the development of skin microecology, reduce skin microbial diversity, and have minimal impact on skin richness during this process. Therefore, boric acid has been an element used for over a century to treat cutaneous *Candida albicans* infections [15]. Natamycin (NAT), produced by various Streptomyces species (Streptomyces natalensis), is a polyene macrolide antibiotic widely used around the world for its broad activity spectrum and resistance-free properties, without any odor or taste [16]. Natamycin disrupts the cell membrane by causing the loss of soluble substances from the cytoplasm. It achieves this by irreversibly binding to fungal ergosterol [17,18]. With its activity, it is effective against most fungi. It is an effective antifungal and antiprotozoal agent used in the treatment of diseases, in the food industry, and as a pesticide [16]. Microextrusion 3D printing is an additive manufacturing technology that enables the controlled layer-by-layer deposition of biomaterials, polymers, or cell-laden solutions [19]. Using a piston or air pressure, the material is extruded through a fine-tipped syringe to form the desired structure. For successful printing, the extrusion speed, air pressure, mechanical deposition rate, and appropriate syringe tip selection are critical. Due to its precision, patient- and tissue-specific designs can be produced [20]. Additionally, it enables the 3D production of personalized medical products and can enhance environmental sustainability by reducing raw material usage and energy consumption. Electrospinning is a technique based on the principle of drawing a polymer solution into micro- or nanoscale fibers under the influence of an electric field between a needle tip and a collector at high voltage. This method enables the production of nanofibers with low polydispersity, high surface area, and controlled porosity [21,22].

In this study, the inner layer (PVA/chitosan/boric acid (PVA/CS/BA)), fabricated by 3D printing, functions as the bioactive and structural core of the wound dressing. Its biological roles include mechanical support and tissue integration, achieved through the PVA–CS matrix, which mimics the viscoelastic behavior of the extracellular matrix [23]. Promotion of wound healing occurs via two key materials such as CS and BA. CS provides intrinsic antibacterial activity, enhances fibroblast adhesion, and accelerates epithelial regeneration [24,25]. BA contributes to macrophage activation, supports cell proliferation, and demonstrates broad-spectrum antimicrobial and anti-inflammatory effects [26,27,28]. The outer layer (electrospun PVA/natamycin (PVA/NAT)), applied by electrospinning, serves as the fungal defense barrier and enhances the dressing’s antimicrobial functionality without compromising the flexibility or gas permeability [29,30]. They provide localized antifungal protection through the incorporation of NAT, a polyene macrolide that selectively binds to ergosterol in fungal membranes, leading to cytoplasmic leakage and cell death [31,32]. They act as a nanofibrous shield that prevents external microbial invasion while allowing for moisture and oxygen exchange, critical for avoiding maceration or anaerobic infection. Enabling sustained drug release through its high surface area and nanoscale porosity, which ensures prolonged therapeutic activity against *Candida albicans*. While the inner PVA/CS/BA layer promotes tissue repair, structural stability, and moisture regulation, the outer PVA/NAT electrospun layer functions as a selective antifungal filter. This dual-layer structure, thus, enables internal biological regeneration and external microbial defense, which are both essential for the treatment of chronic wounds, fungal-infected lesions, or immunocompromised skin environments. This synergistic dual functionality clearly distinguishes our design from conventional mono-layer wound dressings. Unlike prior studies where PVA/CS-based wound dressings are typically fabricated via casting or electrospinning alone [33,34], this study uniquely combines 3D printing to create a structured PVA/CS/BA scaffold, followed by surface functionalization with NAT using electrospinning. This dual-step technique allows for patient-specific and anatomically complex wound dressings and reduces material waste. Moreover, homogeneous antifungal drug distribution is achieved on the surface. The integration of natamycin via electrospinning into a 3D-printed PVA/CS/BA structure represents a first-of-its-kind approach in antifungal wound-dressing design. The inclusion of boric acid into the PVA/CS matrix not only enhances mechanical flexibility and durability but also accelerates wound healing (via macrophage migration and cell proliferation), broad-spectrum antimicrobial activity, and support for skin microecology with minimal disruption. Combining BA in the structural layer and NAT in the functional surface layer forms a dual-acting system with both antibacterial and antifungal properties—offering therapeutic synergy not addressed in earlier studies.

## 2. Experimental

### 2.1. 3D Printing of Wound Dressing with PVA/Chitosan/Boric Acid Ink

A 2% *w*/*v* CS (glacial, 99–100%, Sigma–Aldrich, Darmstadt, Germany) solution was prepared by mixing chitosan in 1% acetic acid (ISOLAB, Eschau, Germany) and stirring with a magnetic stirrer at 30 °C until a homogeneous solution was obtained. A 10% *w*/*v* PVA solution was prepared by mixing PVA (Mw of 85,000–124,000, 99% hydrolyzed, Sigma–Aldrich, Darmstadt, Germany) in distilled water and stirring with a magnetic stirrer at 80 °C until a homogeneous solution was achieved. The PVA and chitosan solutions were then mixed at a 1:1 ratio. Boric acid (AR ≥ 99.5 %, Shanghai Aladdin Biotechnology Co., Shanghai, China) was added to the obtained PVA/CS solution and stirred until homogeneous. The wound dressing was designed using SolidWorks^®^ (3D CAD software, 2022) for 3D printing with a 3D printer (Axolotl, Turkey). For the printing of the solutions, the following parameters were optimized: nozzle size of 21-gauge, infill density of 32%, size set of 30 × 30 mm, print speed of 3 mm/s, and pressure of 15 psi. The 3D printing process was carried out under room temperature conditions.

### 2.2. Coating of 3D Wound Dressings with Natamycin via Electrospinning Method

A 10% *w*/*v* PVA solution was prepared in distilled water at 70 °C. Natamycin was added to the PVA solution at a concentration of 1% of the polymer weight and mixed until homogeneous. The PVA/NAT solution was then loaded into a syringe and placed on the electrospinning pump. During electrospinning, the parameters were optimized as follows: a 25-gauge needle, flow rate of 0.3–0.5 mL/h, high voltage of 15 kV from the power supply, and 12 cm between the collecting plate and the needle tip. After optimization, the PVA/NAT solution was used to coat the PVA/CS/BA 3D structures, which were produced using the electrospinning method with the optimized parameters.

### 2.3. Mechanical Test

The mechanical properties of the samples were assessed through a tensile mechanical test, following the standard procedure outlined in ASTM D882 [3]. The samples were processed into rectangular strips with 100 mm × 20 mm × 0.1 mm dimensions. Tensile strength and strain tests were conducted using a tensile tester (Shimadzu-EZ-LX, Kyoto, Japan) and specialized software. All samples were tested at a 5 mm/min speed until they reached their breaking point. The measurements were taken at room temperature (23 °C).

### 2.4. Chemical Analysis by FTIR

The FT-IR spectroscopy (FT-IR, 4700 Jasco, Tokyo, Japan) was used to analyze the chemical characterization and molecular structure of the produced wound-dressing samples. The spectrum results were analyzed in a scanning range of 4000–400 cm^−1^.

### 2.5. DSC Analysis

The thermal behavior of the samples was analyzed using a differential scanning calorimeter (DSC). The analyses were carried out at a heating rate of 10 °C/min, in the temperature range of 20–250 °C, under continuous nitrogen gas flow using a Perkin-Elmer DSC 4000 device (PerkinElmer, Waltham, MA, USA). The glass-transition temperature (T_g_), melting temperatures (T_m_), and enthalpy of melting (ΔH_m_) of the samples were measured. The degree of crystallinity (X_c_) was calculated using Equation (1), as follows:

(1) $$X_{c}={∆H}_{m}\times {\left({∆H}_{m}^{0}\times w\right)}^{-1}\times 100$$

where w is the weight fraction of the PVA, and ${∆H}_{m}^{0}$ is the enthalpy of melting for 100% crystalline PVA, which was taken as 138.6 J/g [1].

### 2.6. Morphological Analysis by SEM

The surface morphologies of the produced wound-dressing samples were examined using SEM (Hitachi SU3500 T2, Graz, Austria). Before imaging, the surface of the samples was coated with gold for 120 s using a sputter-coating machine (Quorum SC7620, Newhaven, UK). The morphological characteristics, size, and shapes of each sample were analyzed.

### 2.7. XRD Analysis

The X-ray diffraction (XRD) analysis was conducted using a Bruker 8 diffractometer (XRD, Rigaku, RINT 2200 VL, Tokyo, Japan) equipped with Cu Kα radiation (λ = 1.54 Å). The analysis was conducted under an operating voltage of 40 kV and a current of 30 mA. Data were collected over a 2θ range of 0–90° with a step size of 0.02°, ensuring high-resolution measurements. The crystallite sizes were calculated with the help of the Scherrer Formula [35] (Equation (2)).

(2) $$D=\frac{K\lambda }{\beta cos\theta }$$

D = crystallite size;

K = shape factor (typically 0.9 for cubic crystals);

λ = X-ray wavelength (Cu Kα = 1.5406 Å);

β = FWHM of the peak (in radians);

θ = Bragg angle of the peak (in radians).

### 2.8. Evaluation of Natamycin Release

The release of natamycin from the wound dressing samples was evaluated using a UV spectrophotometer. Initially, the samples were incubated in PBS (phosphate-buffered saline) in a circular shaker at 60 rpm and 37 °C. At predetermined time intervals, 3 mL was taken from the solution of each sample, and PBS was added to replace the volume taken. The absorbance of the solutions from each sample was measured at 300 nm for natamycin [36].

### 2.9. Antifungal Test

The antimicrobial effect of the obtained print on the test microorganism was evaluated using the agar diffusion method. *Candida albicans* were cultured on Sabouraud Dextrose Agar for 24 h, and colonies were collected to create a suspension in 5 mL of 0.85% sterile saline. The suspension was adjusted to a transmittance of 75–77% at a wavelength of 530 nm. A measured amount of the suspension was evenly spread over the surface of Mueller–Hinton agar containing 2% glucose and 0.5 µg/mL methylene blue using a sterile spreader, and the surface was allowed to dry. Then, the printed disks (containing 0.1%, 0.5%, and 1% natamycin and a negative control) were placed on the surface of the agar under sterile conditions, and the plates were incubated at 35 °C for 24 h. After the incubation period, the clear zone diameters surrounding the disks were measured in millimeters, and the results were recorded as sensitive, resistant, or dose-dependent sensitive [37].

### 2.10. Cell Viability MTT Test

The samples were separated from the greaseproof paper and placed on 2 × 2 microplates. The L929 mouse fibroblast cells were then seeded into each well of 48-well, flat-bottom microplates at a concentration of 3 × 10^4^ in 500 µL of medium. The cells were incubated at 37 °C for 24 h to allow for cell attachment. After the 24 h incubation period, the entire volume of the culture medium was aspirated. Then, 500 µL of fresh medium containing 5-carboxanilide (MTT) at a concentration of 7.5 mg/mL, along with 0.5 mg/mL of phenazine methosulfate and 2,3-bis-(2-methoxy-4-nitro)-5-sulfophenyl-2H-tetrazolium, was added to each well. The cells were incubated for an additional 4 h at 37 °C. The cell culture medium was used as a negative control. Percent cell viability was calculated by measuring the optical density at 450 nm.

The effect of the different medium volumes (25 mg/mL, 50 mg/mL, 75 mg/mL, and 100 mg/mL) on the cell viability was tested on L929 mouse fibroblast cells seeded into 96-well microplates. The 3D scaffold samples were placed in a cell culture medium for 24 h. After a 24 h incubation period, a standard MTT test was performed, and cell viability was calculated and visualized as a percentage.

### 2.11. Statistical Analysis

All statistical data analyses were conducted using ANOVA with GraphPad Prism, version 8, software (GraphPad Software Inc., San Diego, CA, USA). The values are presented as the mean ± standard deviation (SD), and the statistical differences were analyzed by one-way ANOVA and Tukey and Dunnett multiple comparison tests. In all instances, *p* < 0.0001 was deemed statistically significant.

## 3. Result and Discussion

### 3.1. Mechanical Analysis

**Figure 1:**
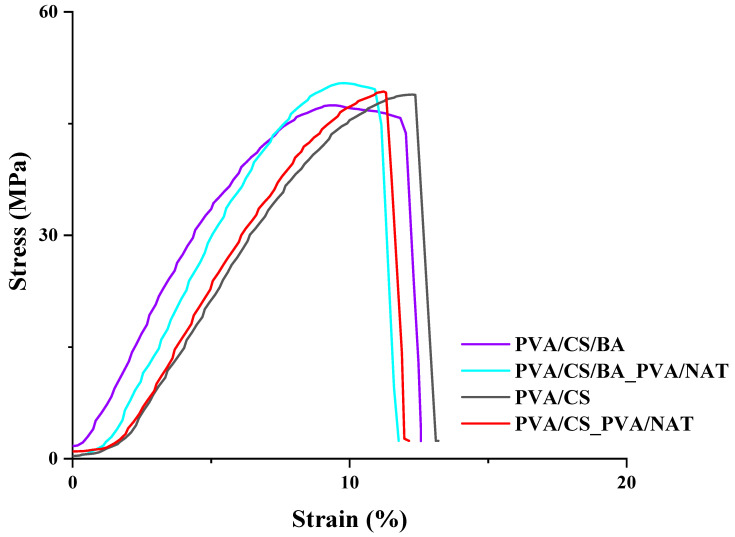
Stress–strain curves of the PVA/CS, PVA/CS/BA, PVA/CS_PVA/NAT, and PVA/CS/BA_PVA/NAT 3D-fiber composite scaffolds.

Figure 1 presents the stress (stress, MPa)–strain (strain, %) curves to compare the mechanical properties of the different composite materials. The material codes on the graph are PVA/CS, PVA/CS/BA, PVA/CS_PVA/NAT, and PVA/CS/BA_PVA/NAT. The mechanical behavior of each material is quantitatively compared, especially in terms of the maximum tensile strength and elongation at break. The PVA/CS/BA sample reached a maximum tensile strength of around 52 MPa and an elongation at break of around 11%. This indicates that boric acid (BA)-doping contributes significantly to the mechanical strength of the PVA/CS matrix. The structure formed by the hydrogen bonds of the PVA and CS became more cross-linked and rigid in the presence of boric acid, resulting in high tensile values. The PVA/CS/BA_PVA/NAT sample is the composite with the highest maximum tensile value on the graph, with a peak of around 55 MPa. The elongation at break is also in the widest range compared with the other groups, around 12%. This result reveals that the synergy of both boric acid and natural additive (NAT) and the dual-layer structure (PVA/CS/BA_PVA/NAT) enhances both the strength and flexibility of the composite. This structure exhibits the highest mechanical performance. The PVA/CS composite shows a maximum tensile strength of about 48 MPa and elongation at break is around 11%. This value indicates that the pure PVA/CS blend without BA additive exhibits relatively lower mechanical properties. In the case of adding boric acid to the structure (PVA/CS/BA), a tensile increase of about 4 MPa was observed. This confirms that boric acid increases the tensile strength by increasing the crosslink density in the matrix. The PVA/CS/BA_PVA/NAT sample shows a maximum tensile strength of about 50 MPa, while the elongation at break is in the range of 10–11%. The naturally doped PVA structure offers higher strength than the CS doping alone but is limited in terms of flexibility. This suggests that the particle distribution of the natural additive and its interaction with the matrix has a tensile strength-enhancing but flexibility-limiting effect.

Overall, the PVA/CS/BA_PVA/NAT composite stands out as the most superior system in terms of mechanical performance, providing both the highest tensile strength and the largest deformation capacity. Compared to other systems, it has a more balanced and optimized structure in terms of both strength and flexibility. These findings show that multi-component hybrid systems provide a significant improvement in mechanical properties.

The PVA/CS/BA fiber composite is one of the high stiffness systems, with an elastic modulus of 714.59 ± 12.42 MPa. This structure has a maximum tensile strength of 47.45 ± 1.26 MPa and an elongation at break of 12.57 ± 1.38%. These values indicate that boric acid increases the hydrogen bonds in the PVA/CS matrix, giving the structure a significant stiffness. At the same time, this system has a sufficient deformation capacity in terms of flexibility, which positions it as a system with a balanced mechanical performance. The PVA/CS/BA_PVA/NAT 3D-fiber composite has the highest elastic modulus among all samples, with a value of 763.04 ± 14.54 MPa, reflecting the highest stiffness in the structure (Table 1). The maximum tensile strength was also the highest at 50.45 ± 2.58 MPa. However, the elongation at break remains moderate at 11.77 ± 0.49%, indicating that the system is relatively less flexible despite its high strength. The NAT additive here limited the deformation capacity of the matrix, probably due to the inorganic filler effect, while simultaneously increasing the stiffness and strength. Thanks to the dual-phase structure, an optimized mechanical structure was obtained by taking advantage of both the PVA/CS/BA’s and PVA/NAT’s properties. The PVA/CS fiber composite exhibits the lowest stiffness among the four systems, with an elastic modulus value of 628.36 ± 10.11 MPa. However, the maximum tensile strength is 48.89 ± 2.20 MPa, which is quite significant for a base matrix without BA contribution. The most striking feature of this system is the highest elongation at break of 13.20 ± 1.22%. This shows that a more flexible structure is formed due to the low crosslinking density, but this structure is limited in terms of stiffness and strength. Therefore, the PVA/CS structure may be advantageous for softer and more flexible applications. The PVA/NAT fiber composite exhibits a moderate stiffness with an elastic modulus of 653.78 ± 9.94 MPa and reflects high strength with a maximum tensile strength of 49.30 ± 2.35 MPa. The elongation at break was 12.14 ± 1.05%. These properties indicate that the NAT additive together with PVA improves the mechanical properties in general but has a limited effect in terms of flexibility. Nevertheless, the system offers a stable performance in terms of both stiffness and strength.

### 3.2. FTIR Analysis of Composite Nanofibers

**Figure 2:**
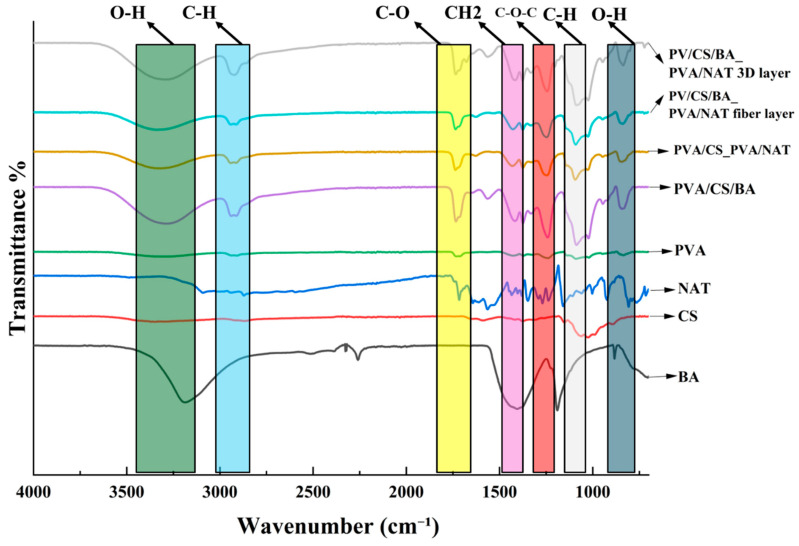
FTIR structures of PVA, CS, BA, NAT, PVA/CS, PVA/CS_PVA/NAT, PVA/CS/BA, and PVA/CS/BA_PVA/NAT 3D-fiber composite scaffolds.

The FTIR spectra of the PVA/CS/BA_PVA/NAT 3D-fiber composites are shown in Figure 2. The stretching vibrations of the hydrogen-bonded -OH groups are responsible for the broad band observed at 3313 cm⁻^1^ in the PVA [38]. The interactions between the PVA chains in the presence of BA are shown by the minor shift and decreased intensity of this band in the PVA/BA spectra. The band at 2930 cm⁻^1^, present in both the PVA and PVA/BA, corresponds to the C-H stretching vibrations [39]. The absence of significant intensity changes suggests that boron incorporation does not affect the C-H bonds. Carbonyl (C=O) stretching vibrations are exhibited by a strong band in the PVA/BA spectra at 1726 cm⁻^1^ [40]. The absence or weakness of this band in the PVA nanofiber indicates that boron doping into the PVA structure led to the formation of carbonyl groups. The -CH₂ bending and C-O-C asymmetric stretching vibrations in the PVA are represented by the bands at 1438 cm⁻^1^ and 1247 cm⁻^1^ [41]. The effect of the NAT on the PVA nanofiber is evident in the variations in the intensity and position of these bands in the PVA/NAT composite nanofiber. Additionally, C-O stretching vibrations in the PVA are demonstrated by bands at 1090 cm⁻^1^ [42]. The changes in these bands in the PVA/NAT spectra further demonstrate the effects of boron additions on the C-O structure.

### 3.3. DSC

**Figure 3:**
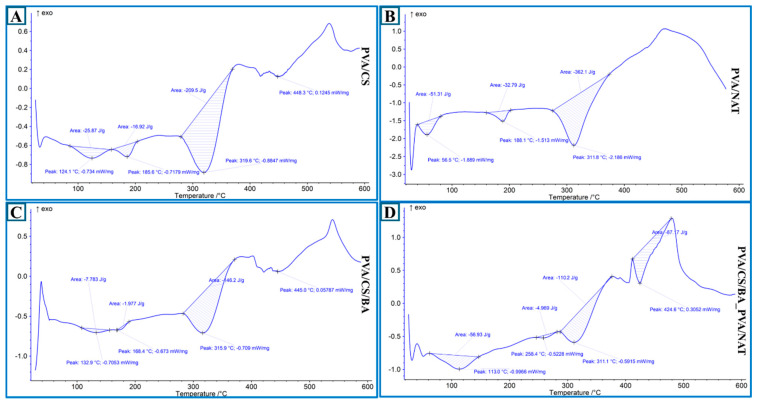
DSC analysis: (**A**) PVA/CS; (**B**) PVA/NAT; (**C**) PVA/CS/BA; (**D**) PVA/CS/BA_PVA/NAT 3D-fiber composite scaffolds.

The DSC (differential scanning calorimetry) curves reveal the thermal behavior of the different PVA-based composite materials. In the graphs, the parameters, such as thermal transition temperatures (especially glass transition and decomposition points), exothermic/endothermic reactions, and heat flux (mW/mg), are evaluated (Figure 3).

The PVA/CS (Figure 3A) fiber composite exhibits three distinct endothermic transitions. The first peak occurs at 124.1 °C with a heat flux of −0.734 mW/mg, probably reflecting the physical water loss and glass transition of the PVA and CS. The second endothermic event is observed at 185.5 °C, indicating the melting of the semi-crystalline regions in the structure. The main degradation peak occurred at 319.6 °C with an energy of about 0.8647 mW/mg, which is the thermal stability limit of the composite. This peak is followed by a second large exothermic event at 448.3 °C, which probably corresponds to the onset of carbonization. The total energy absorption is around 200.5 µJ, indicating that the structure is moderately resistant to heat. In the PVA/NAT composite (Figure 3B), the thermal events start with a low enthalpy peak at 56.5 °C (probably moisture removal) and a higher energy endothermic transition with −1.513 mW/mg at 188.1 °C. The most striking point is the main decomposition peak at 311.8 °C, which has a very strong energy transfer of about 2.166 mW/mg. This suggests that the NAT doping accelerates the degradation within the matrix, resulting in a more pronounced energy output. The integrated area (energy absorption) is quite high at 392.1 µJ, indicating that thermal transitions are more intense and the structure is more active against the thermal effect. The PVA/CS/BA (Figure 3C) fiber composite shows a glass transition starting at 132.9 °C and −0.7053 mW/mg. In this system, more stable behavior against heat is observed due to the BA doping [42,43]. The main decomposition peak is at 315.9 °C and 0.770 mW/mg, with a thermal stability range extending up to 445.0 °C. The energy absorption of this structure is 149.2 µJ. The increased crosslink formation of BA in the structure slightly increased the thermal stability, while the overall reactions to heat were more limited (lower enthalpy). The PVA/CS/BA_PVA/NAT (Figure 3D) 3D-fiber composite exhibits a more complex thermal behavior due to the multiphase structure. The first significant transition occurs at 113 °C with −0.9666 mW/mg, indicating the removal of moisture and some low-molecular-weight components from the structure. The main decay starts at 311.1 °C (−0.5015 mW/mg) and the second major peak continues at 424.6 °C with −0.3052 mW/mg. The largest enthalpy change is observed at 237.1 µJ. This high energy absorption indicates that the synergistic contributions in the structure (BA and NAT) lead to higher energy requirements in thermal transitions and, therefore, the system becomes more reactive to heat [44,45]. This structure stands out as the composite that is most reactive to heat and exhibits the most pronounced thermal phenomena.

The highest stability in terms of thermal strength is observed in the PVA/CS/BA sample, while the highest energy absorption and thermal activity belongs to the PVA/CS/BA_PVA/NAT system. This indicates that the multiphase hybrid structure presents both a stable and reactive thermal profile. In contrast, the PVA/CS system shows a simple and controlled thermal behavior, while the PVA/NAT exhibits degradation at lower temperatures but with high heat flux.

### 3.4. Morphological Investigation of Composite Nanofibers

The morphology and fiber diameter distribution of the PVA/CS, PVA/CS_PVA/NAT, and PVA/CS/BA fiber composites and PVA/CS/BA_PVA/NAT 3D-fiber structures were evaluated by scanning electron microscopy (SEM) images and statistical histogram analysis. Each of these structures was obtained by electrospinning, and the differences in their composition show significant effects on fiber diameter and distribution uniformity.

**Figure 4:**
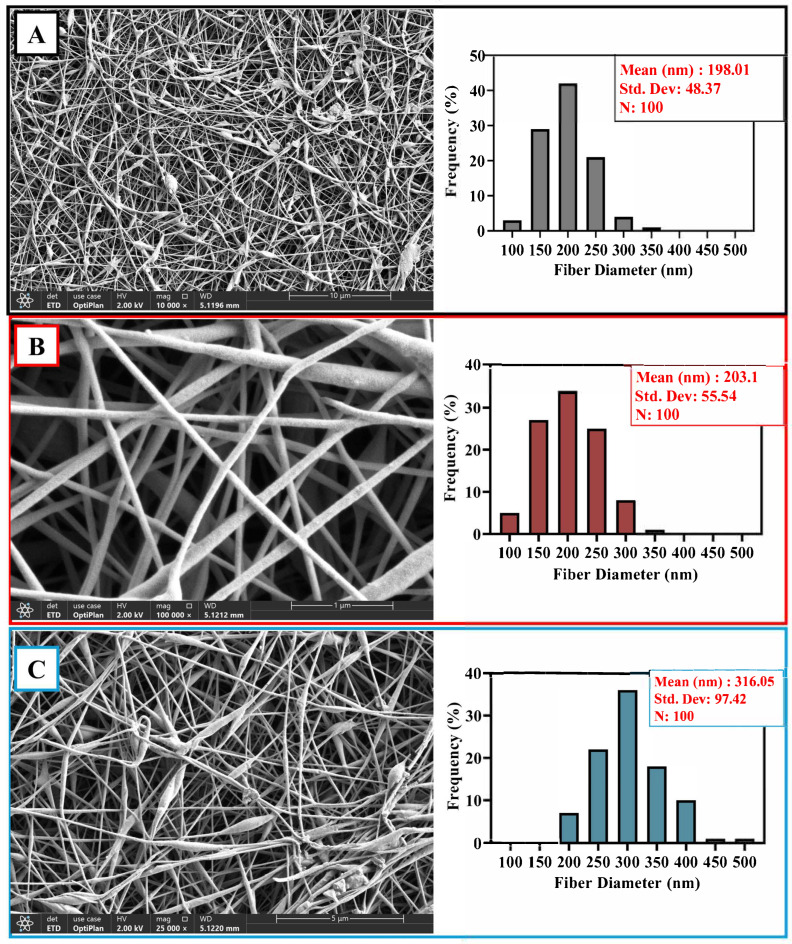
SEM images and histograms: (**A**) PVA/CS; (**B**) PVA/CS_PVA/NAT; (**C**) PVA/CS/BA composite fibers.

The image of the PVA/CS composite shows a very uniform and homogeneous nanofiber network. The fibers are generally parallel to each other, and there are no obvious agglomerations or structural distortions. According to histogram analysis, the average fiber diameter is 198.01 ± 48.37 nm, showing a narrow distribution (Figure 4A). This indicates that the electrospinning conditions are controlled, and the resulting fiber mat is homogeneous. Most of the fiber diameter distribution is concentrated in the 150–250 nm range. This range can provide favorable surface properties, especially for cell adhesion and proliferation [46,47].

The PVA/NAT composite contains slightly thicker fibers, and the morphological structure is still regular (Figure 4B). Slightly increased attachment points between fibers and more pronounced orientations are observed. The histogram shows a mean fiber diameter of 203.1 ± 55.54 nm, which is slightly wider than in the PVA/CS structure but still shows a controlled distribution [29,48]. The fiber diameters were again predominantly concentrated between 150 and 300 nm, suggesting that the BA additive affected the rheology of the solution, leading to an increase in fiber diameter. This increase in thickness may contribute to the improvement of mechanical strength.

The PVA/CS/BA composite has the most heterogeneous structure. In the SEM images, it is observed that the fibers are highly irregular in terms of both the diameter and density, and, in some areas, the fibers stick together or form bundles (Figure 4C). According to the histogram analysis, the average fiber diameter is 316.05 ± 97.42 nm, with a very high standard deviation of nm, indicating that the distribution is wide [42]. The fiber diameters spread from 150 nm to 450 nm and were particularly concentrated in the range of 250–350 nm. These results reflect the morphological diversity of the structure obtained with the combination of two different solutions (PVA/CS/BA and PVA/NAT). The fact that the fibers are thicker and structurally more complex may increase the porosity of the material, which could be advantageous for biomedical applications, such as cell penetration or fluid migration. However, this irregularity can be limited in terms of fiber integrity and long-term mechanical stability.

Overall, the PVA/CS structure presented the most homogeneous and fine fibers, while gradual increases in the fiber diameter and widening of its distribution were observed with the BA and NAT additives. These results clearly demonstrate the influence of composite ingredients on the electrospinning process and suggest that the desired morphological properties for specific biomedical applications can be achieved through compositional optimization.

**Figure 5:**
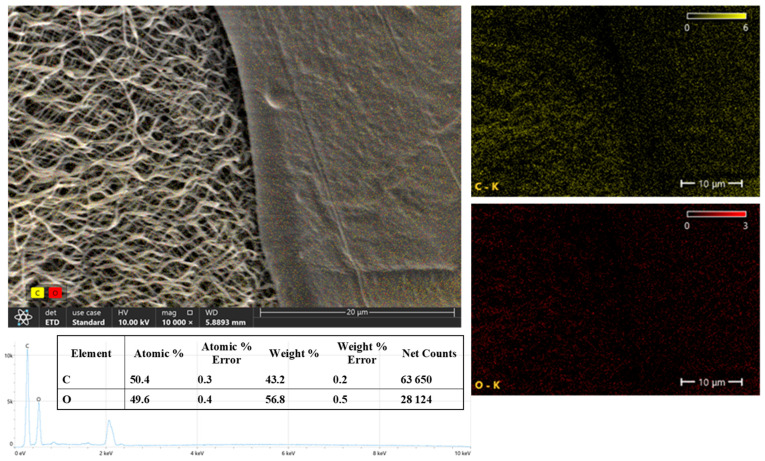
SEM-EDS map of the PVA/CS/BA_PVA/NAT 3D-fiber scaffold: fiber layer images.

According to the quantitative EDS analysis, carbon was atomically 50.4% and oxygen 49.6%. By weight, the carbon was 43.2% and oxygen 56.8% (Figure 5). This elemental composition indicates that the structure is largely of organic origin and contains dense oxygen-containing groups (e.g., hydroxyl or carboxyl). This suggests that the composite has potentially high surface energy and good biocompatibility properties. In addition, it is determined that the surface in combination with the fibrous structure can offer a large active area and, therefore, may be particularly advantageous in biosensor applications, cell culture supports, or tissue-engineering scaffolds.

**Figure 6:**
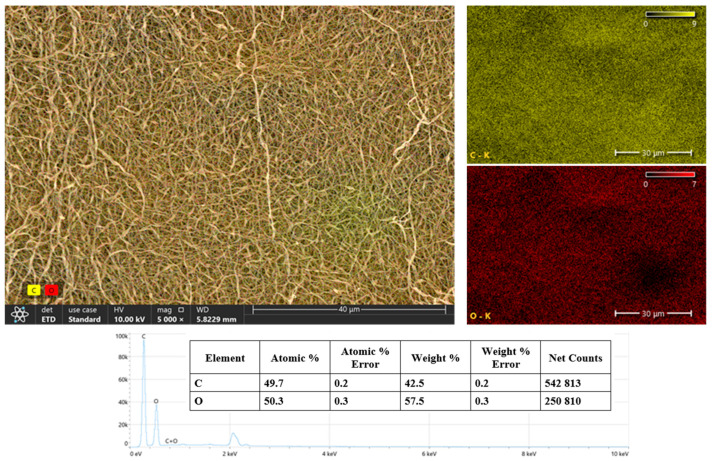
SEM-EDS map of the PVA/CS/BA_PVA/NAT 3D-fiber scaffold: 3D layer images.

The SEM imaging results, supported by an EDS analysis, reveal that the surface morphology of the sample shows a homogeneous distribution and contains prominent crack-like structures. Elemental mapping data show that the carbon (C) and oxygen (O) elements are distributed over the entire surface and show a homogeneous distribution of these elements. According to the quantitative EDS results, the atomic percentages of carbon and oxygen were 49.7% and 50.3%, respectively, indicating that the sample is largely composed of carbon and oxygen. According to the weight ratios, the carbon was 42.5% and the oxygen was 57.5% (Figure 6). These results suggest that the composite is of an organic origin and probably contains oxygen-containing functional groups such as hydroxyl, carboxyl, or ester. Such functional groups enhance the material’s properties such as biocompatibility and hydrophilicity, supporting its usability, especially in biomedical applications (e.g., wound dressings, tissue scaffolds, or biosensors).

### 3.5. XRD Analysis of the Composite Nanofibers

**Figure 7:**
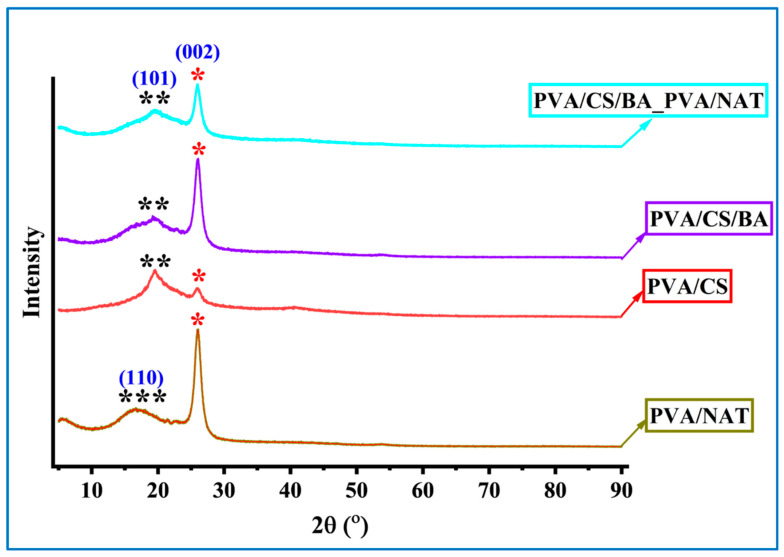
XRD analysis of the PVA/CS, PVA/NAT, PVA/CS/BA, and PVA/CS/BA_PVA/NAT 3D-fiber composite scaffolds (miller indices; *: 002, **: 101, ***: 110).

The XRD (X-ray Diffraction) analysis graph allows for a comparative evaluation of the crystalline and amorphous structural properties of four different composite systems. The diffraction patterns of these PVA-based composites (Figure 7) clearly reveal the influence of interactions between the components on the degree of crystallinity and differences in structural regularity.

In the PVA/NAT sample, the prominent diffraction peaks observed at 2θ ≈ 19.5° and 29.4° represent the (110) plane in the NAT structure and characteristic reflection of the crystalline structure of PVA, respectively. The broad peak at 19.5° (***) reflects the crystalline nature of the NAT, while the sharp peak at about 29.4° shows the limited crystallinity of the PVA [49,50]. This example suggests that crystalline properties are preserved by homogeneous dispersion of the components and the contribution of the amorphous phase is minimal. Moving to the PVA/CS formulation, a more diffuse amorphous peak around 2θ ≈ 20° marked (**) is observed, indicating the semi-crystalline nature of the chitin derivative chitosan and its interactions with the PVA. In the PVA/CS sample, the crystalline peak of PVA at around 29.4° is noticeably attenuated (*) [51,52]. This indicates that physical interactions between the PVA and CS, such as hydrogen bonding, disrupt the crystalline regions and the structure becomes more amorphous. In the case of the PVA/CS/BA, the crystalline peaks are further suppressed with the addition of BA. The broadened amorphous signals (**) in the range 2θ ≈ 19–21° indicate a decrease in ordered structures and an increase in more disordered, amorphous regions as a result of doping of the polymer chains with BA. The characteristic crystalline peak of PVA at 29.4° (marked with *) has a lower intensity compared with the previous samples, confirming the decrease in crystallinity. This can be explained by the bioactive character that BA adds to the structure.

Finally, in the PVA/CS/BA_PVA/NAT composite, the combination of all components (PVA, CS, BA, and NAT) shows both amorphous and crystalline characteristics. The prominent peaks with indexes at 2θ ≈ 19.5° (101) and 29.4° (002) indicate that the crystalline contributions of both the NAT and PVA are again evident [42,53]. This suggests that the contribution of the NAT in the system favors crystalline arrangements, partially offsetting the influence of the amorphous structures formed by BA and CS. At the same time, the persistence of broad (**) signals corresponding to the amorphous structure indicates that the structure has the character of a heterogeneous crystalline–amorphous composite.

In conclusion, the XRD data clearly reveals the effects of different additives on the crystalline nature of the PVA matrix. The crystalline structure is preserved to the highest extent in the PVA/NAT system, while the amorphous character is dominant in the PVA/CS system. BA doping enables partial regularization, while the multiphase system PVA/CS/BA_PVA/NAT offers a complex but balanced combination of crystallinity/amorphous structure. This structural diversity consists of variations in both mechanical and thermal properties.

### 3.6. Release Kinetics Study

**Figure 8:**
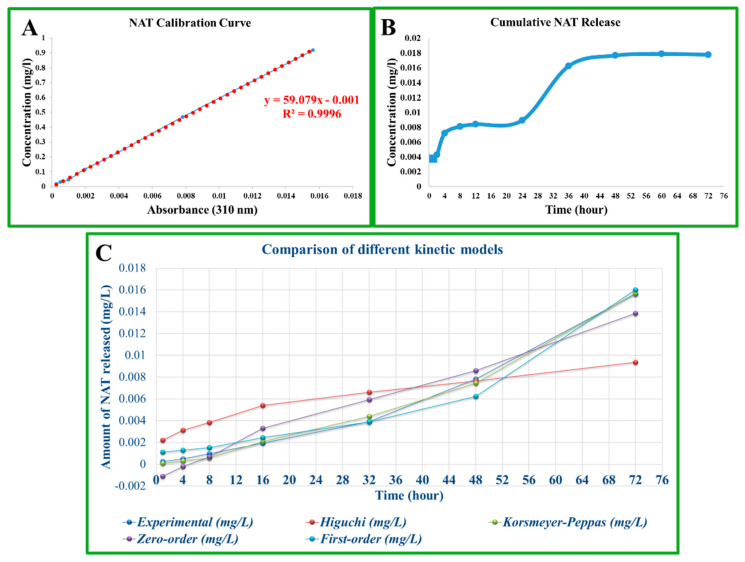
(**A**) NAT calibration curve and (**B**) NAT cumulative release graph, (**C**) comparison of different kinetic models.

The NAT calibration curve in Figure 8A shows the NAT concentrations versus absorbance values obtained at a 310 nm wavelength. The equation obtained from the linear regression analysis is y = 59.079x − 0.001, and the coefficient of determination is R^2^ = 0.9996. This high R^2^ value indicates that the measurement system is highly accurate and reliable. The calibration curve obtained confirms that the amount of NAT can be accurately determined by UV-Vis spectrophotometry. Figure 8B shows the cumulative NAT release over time. When the release profile is evaluated, a rapid release phase (burst release) is observed in the first 8 h. Following this phase, a slower and more controlled release was observed from about 24 h onwards, reaching a plateau at 72 h. This suggests that the system works with a fast and then slow-release mechanism and probably realizes an in-matrix diffusion-controlled release. In conclusion, these data show that the developed composite structure has potential for controlled drug release systems [36,54]. In particular, a rapid initial dose followed by sustained release is advantageous in terms of prolonging the therapeutic effect.

The Higuchi model is generally valid in diffusion-controlled release systems. If the curve in Figure 8B shows the linearity versus the square root time, especially in the 4–72 h range, it can be said that the release is consistent with the Higuchi model. This suggests that the NAT is released from the surface and interior of the carrier matrix by Fick-type diffusion [54,55]. The Korsmeyer–Peppas model is particularly suitable for the first 60% of the release data. The value of *n* determines the release mechanism. If *n* ≈ 0.5 in this model, it is consistent with Fickian diffusion; if *n* is between 0.5 and 0.89, this indicates both diffusion and anomalous transport associated with swelling/erosion of the matrix. The zero-order model release rate is time-independent, i.e., a constant rate of release. Since the release profile in Figure 8B does not show a constant slope; it does not seem to fit well with zero-order kinetics. The first-order model decreases with time depending on the concentration of the remaining drug. The shape of the curve in Figure 8B may partially fit this model. As observed in Figure 8B, The rapid release was in the first 30 min, followed by a tendency to plateau at 36–72 h; this profile can most likely be explained by the Korsmeyer–Peppas model, and it can be said that the release shows anomalous (non-Fickian) diffusion properties (Figure 8C). As a result, the Korsmeyer–Peppas model is the model with the highest fit for the NAT release (R^2^ = 0.997) (Table 2). An *n* > 1 indicates that the release proceeds by super-accidental diffusion or swelling-controlled mechanisms. The first-order model also exhibited a high R^2^ value, suggesting that the release occurs at a decreasing rate with the concentration. The Higuchi model is inadequate for these data (R^2^ < 0.6).

### 3.7. Antimicrobial Tests

In this study, the antifungal activities of various biopolymer-based formulations against *Candida albicans* were evaluated (Table 3). No inhibition zone was observed in the control group containing only polyvinyl alcohol (PVA) (0 ± 0 mm), indicating that PVA alone does not possess inherent antifungal properties. In contrast, the addition of chitosan to the PVA matrix significantly increased the antifungal activity (1.05 ± 0.12 mm, *p* < 0.05). This finding is consistent with previous reports showing the antimicrobial potential of chitosan. Rabea et al. [56] reported that chitosan disrupts the fungal cell morphology by increasing the membrane permeability through electrostatic interactions. Further enhancement was observed with the incorporation of boric acid (1.25 ± 0.08 mm), likely due to its fungistatic activity by reducing intracellular ATP levels in *Candida* species [57]. The inhibition zone diameters of 1.43 ± 0.08 cm (14.3 mm) for the PVA/CS_PVA/NAT and 1.64 ± 0.13 cm (16.4 mm) for the PVA/CS/BA_PVA/NAT indicate superior antifungal performances, likely due to the synergistic effect of chitosan, boric acid, and natamycin in a nanofiber-based matrix. This suggests that our hybrid formulations may offer enhanced local drug delivery and prolonged antifungal action compared to nanoparticulate systems alone. The formulations containing natamycin exhibited the highest antifungal effects. The PVA/CS_PVA/NAT composite (1.43 ± 0.08 mm) and PVA/CS/BA_PVA/NAT (1.64 ± 0.13 mm) showed significantly larger inhibition zones compared to other groups (*p* < 0.05). Natamycin is known to exert its antifungal action by binding to ergosterol in the fungal cell membrane, disrupting membrane integrity and causing cytoplasmic leakage [31]. These findings suggest a synergistic effect when natamycin is combined with biopolymers. Our results are also comparable to previously reported values in the literature. Liu et al. [58] developed natamycin-loaded chitosan nanoparticles and reported inhibition zones of 11.32 ± 0.18 mm at 24 h against *Candida albicans*. The larger inhibition zones observed in our formulations (14.3 mm and 16.4 mm, respectively) may be attributed to the nanofibrous structure providing improved surface contact, sustained release, and combined antifungal mechanisms.

The observed antifungal activity can be attributed to the complementary mechanisms of action of the chitosan, boric acid, and natamycin. Chitosan is known to interact electrostatically with negatively charged fungal cell membranes, increasing the membrane permeability and disrupting the ionic homeostasis. Additionally, low-molecular-weight chitosan derivatives may penetrate the cell and interfere with DNA and protein synthesis [56]. The contribution of boric acid to the antifungal activity is likely related to its inhibitory effects on enzymes involved in cell wall synthesis and its interference with cellular energy metabolism. It has been shown to reduce intracellular ATP levels in Candida albicans, thereby exerting a fungistatic effect [57]. Natamycin exerts its antifungal effect by specifically binding to ergosterol, a key component of fungal cell membranes, leading to membrane destabilization and cell lysis [59]. Taken together, the combination of these agents targets multiple cellular structures and pathways, resulting in a synergistic antifungal effect against *Candida albicans*.

### 3.8. Cell Study

The findings from the MTT assay are essential for evaluating the biocompatibility of the PVA/CS-based 3D-fiber composites intended for tissue-engineering and wound-dressing applications. The materials must exhibit minimal toxicity and high cell viability for these purposes. Cell viability rates exceeding 98% indicate that these materials are suitable for sensitive applications such as wound dressings [38].

**Figure 9:**
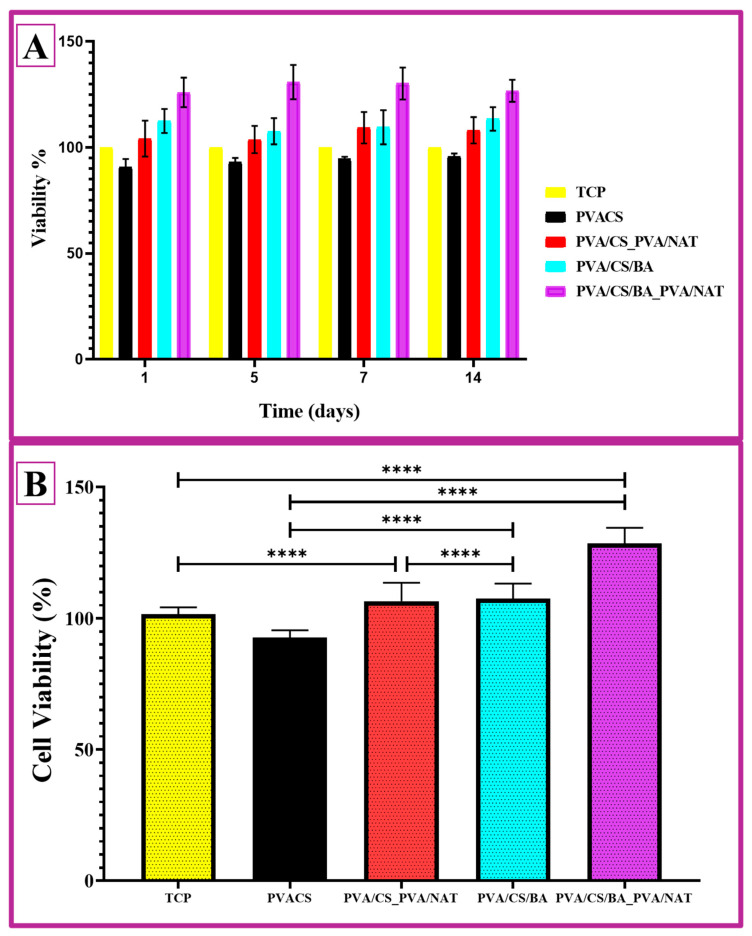
(**A**) L929 fibroblast cell viability of the PVA/CS, PVA/CS_PVA/NAT, PVA/CS/BA, and PVA/CS/BA_PVA/NAT composite 3D-fiber; (**B**) cell viability by the MTT method (****, mean ± SD values of n = 4 independent experiments; *p* < 0.0001 compared to the control. The data were analyzed using a one-way analysis of the ANOVA with Tukey’s multiple comparison test).

The cell viability data presented here provides a time-dependent and comparative assessment of the biocompatibility of the four different PVA-based composites studied. The time series of cell viability in Figure 9A includes measurements on days 1, 5, 7, and 14, and Figure 9B presents the average cell viability (%) based on the statistical significance. The PVA/CS fiber composite shows the lowest cell viability value compared to the other systems. At all of the time points, the viability rate remained at around 90%, suggesting that the construct is acceptable in terms of the basic biocompatibility but weaker compared to the other systems. The possible reason could be the moderate biocompatibility of chitosan and the limitation of the surface interaction due to its high density of hydrogen bonds. The PVA/NAT fiber composite, on the other hand, exhibited a cell survival rate of over 100% from day one. The NAT additive may have made the surface morphology more favorable for cell adhesion; in addition, the biologically inert and porous structure of the NAT may have facilitated cell adhesion and proliferation. After 14 days, the viability level remained high, indicating the long-term biocompatibility of the material. The PVA/CS/BA composite exhibits a significant improvement over the PVA/CS system due to the effect of the BA. The cell survival rate was above 100% at all time points and showed an increasing trend from day 5 onwards. This can be explained by the BA increasing the surface hydrophilicity and structural flexibility to promote cell proliferation. The PVA/CS/BA_PVA/NAT 3D-fiber composite stands out as the structure with the highest cell viability among all of the groups. Starting at around 110% on day 1, the survival rate approaches 130% by the end of day 14, indicating that both rapid cell adhesion and proliferation are effectively supported. The multiphase character and porous morphology of the structure may have enabled the cells to interact more effectively with the surface. At the same time, this hybrid structure combines both the biological inertness of the PVA/NAT and the binding ability of the BA, providing a very favorable microenvironment for cells [60].

The statistical significance levels (**** *p* < 0.0001) indicate that the differences between the PVA/CS and the other three groups are particularly pronounced and significant (Figure 9B). The superiority of the PVA/CS/BA_PVA/NAT 3D-fiber composite over all of the groups indicates that this composite could be a strong candidate for applications such as tissue engineering, wound dressings, or biomedical implants.

## 4. Conclusions

In this study, a series of biopolymer-based composite nanofibers were successfully developed using electrospinning and 3D bioprinting strategies, combining polyvinyl alcohol (PVA), chitosan (CS), boric acid (BA), and a natural antifungal agent NAT. The most complex formulation, PVA/CS/BA_PVA/NAT, was designed as a dual-layer structure via 3D bioprinting, enabling spatially controlled incorporation of functional additives within a single-composite system. Mechanical characterization revealed that the dual-layer PVA/CS/BA_PVA/NAT system exhibited the highest elastic modulus (763.04 ± 14.54 MPa) and tensile strength (50.45 ± 2.58 MPa), indicating excellent load-bearing potential and enhanced structural stability compared to the single-layer analogues. The SEM and fiber diameter distribution analyses confirmed the formation of uniform, bead-free, and thinner nanofibers in the BA-containing structures, contributing to both mechanical reinforcement and potential for controlled diffusion. The drug release experiments demonstrated a sustained NAT release profile over a 90 min period. Kinetic modeling revealed a dominant Higuchi mechanism, supported by a Fickian-diffusion profile from Korsmeyer–Peppas modeling, which collectively suggest a matrix-controlled passive diffusion process. These release dynamics were especially effective in the dual layer structure, enabling regulated therapeutic delivery. Furthermore, antifungal testing against *Candida albicans* revealed a pronounced inhibitory effect by the PVA/CS/BA_PVA/NAT system (1.64 ± 0.13 cm inhibition zone), significantly exceeding the activity of individual component systems. This synergistic effect between the BA and NAT within the 3D-fiber composite dual layer demonstrates the added functional value of architectural design in bioactive material systems. Overall, the combination of improved mechanical resilience, tailored fiber morphology, controlled drug release, and superior antifungal efficacy positions the PVA/CS/BA_PVA/NAT nanofiber composite as a strong candidate for future applications in wound dressings, transdermal therapies, and antifungal biomaterials.

## Figures and Tables

**Table 1:** E modulus (MPa), ultimate stress (MPa), and strain at break (%) of PVA/CS, PVA/NAT, PVA/CS/BA, and PVA/CS/BA_PVA/NAT 3D-fiber composite scaffolds.

	E Modulus (MPa)	Ultimate Stress (MPa)	Strain at Break (%)
**PVA/CS/BA**	714.59 ± 12.42	47.45 ± 1.26	12.57 ± 1.38
**PVA/CS/BA_PVA/NAT**	763.04 ± 14.54	50.45 ± 2.58	11.77 ± 0.49
**PVA/CS**	628.36 ± 10.11	48.89 ± 2.20	13.20 ± 1.22
**PVA/CS_PVA/NAT**	653.78 ± 9.94	49.30 ± 2.35	12.14 ± 1.05

**Table 2:** Comparison of the fit of Higuchi, Korsmeyer–Peppas, zero-order, and first-order models of the NAT release kinetics according to the R^2^ values.

Model	Formula	Parameters	R^2^ Value	Comment
**Higuchi**	*f*_t_ = Q = K_H_ t^1/2^	k_H_ = 0.00099	0.589	Weak fit—the Higuchi model does not seem to fit the release data.
**Korsmeyer–Peppas**	Mt/M∞ = K_r_t^n^ + *b*	k = 3.98 × 10^−6^, n = 1.84	0.997	Very strong fit—the release can be explained by anomalous diffusion (n > 1).
**Zero-order**	*C_t_* = *C*_0_ + *K*_0_ *t*	k_0_ = 0.00018, Q_0_ = −0.00198	0.936	Good agreement—the release may have occurred at a constant rate.
**First-order**	log Q_1_ = log Q_0_ + K_1_t/2.303	C_0_ = 0.00095, k_1_ = 0.0314	0.976	Good agreement—the release may have occurred with a logarithmically decreasing rate.

**Table 3:** Mean inhibition zone diameters (mm ± SD) of different formulations against *Candida albicans*.

PVA	PVA/CS	PVA/CS/BA	PVA/CS_ PVA/NAT	PVA/CS/BA_PVA/NAT
0 ± 0 ^E^	1.05 ± 0.12 ^D^	1.25 ± 0.08 ^C^	1.43 ± 0.08 ^B^	1.64 ± 0.13 ^A^

A–E: Statistical analysis was performed using one-way ANOVA followed by Tukey’s post hoc test (*p* < 0.05). Groups not sharing the same letter are significantly different. Different letters indicate statistically significant differences among groups (Tukey’s HSD, *p* < 0.05).

## Data Availability

The authors confirm that the data supporting the findings of this study are available within the article. The raw data that support the findings of this study are available from the corresponding author upon reasonable request.
